# Barriers and facilitating factors of care coordination for children with spinal muscular atrophy type I and II from the caregivers' perspective: an interview study

**DOI:** 10.1186/s13023-023-02739-w

**Published:** 2023-06-02

**Authors:** Jana Willems, Isabella Bablok, Erik Farin-Glattacker, Thorsten Langer

**Affiliations:** 1 Section of Health Care Research and Rehabilitation Research, Institute of Medical Biometry and Statistics, Faculty of Medicine and Medical Center, University of Freiburg, Hugstetter Straße 49, 79106 Freiburg, Germany; 2 Department of Neuropediatrics and Muscle Disorders, Center for Pediatrics, Faculty of Medicine, University of Freiburg, Freiburg, Germany

**Keywords:** Children with medical complexity, Spinal muscular atrophy, Rare diseases, Care coordination, Semi-structured interviews

## Abstract

**Background:**

Children with medical complexity (CMC) require long-term care accompanied by different health- and social care professionals. Depending on the severity of the chronic condition, caregivers spend a lot of time coordinating appointments, communicating between providers, clarifying social legal issues, and more. Effective care coordination is seen as key to addressing the fragmented care that CMC and their families often face. Spinal muscular atrophy (SMA) is a rare genetic, neuromuscular disease which care involves drug therapy and supportive treatment. We examined the care coordination experiences through a qualitative interview analysis of n = 21 interviews with caregivers of children with SMA I or SMA II.

**Results:**

The code system consists of 7 codes and 12 sub-codes. “Disease and coordination management of the caregivers” describes the management of coordination-related illness demands. “General conditions of care” include enduring organizational aspects of the care network. “Expertise and skills” refers to both parent and professional expertise. “Coordination structure” describes the assessment of existing coordination mechanisms as well as the need for new ones. “Information exchange” defines the information exchange between professionals and parents as well as the exchange of parents among themselves and the perceived exchange between professionals. “Role distribution in care coordination” summarizes parents' “distribution” of coordinative roles among care network actors (including their own). “Quality of relationship” describes the perceived quality of the relationship between professionals and family.

**Conclusion:**

Care coordination is influenced peripherally (e.g., by general conditions of care) and directly (e.g., by coordination mechanisms, interaction in the care network). Access to care coordination appears to be dependent on family circumstances, geographic location, and institutional affiliation. Previous coordination mechanisms were often unstructured and informal. Care coordination is frequently in the hands of caregivers mainly as the care network’s interface. Coordination is necessary and must be addressed on an individual basis of existing resources and family barriers. Existing coordination mechanisms in the context of other chronic conditions could also work for SMA. Regular assessments, centralized shared care pathways, and staff training and empowerment of families for self-management should be central components of all coordination models.

*Trial registration*: German Clinical Trials Register (DRKS): DRKS00018778; Trial registration date 05. December 2019—Retrospectively registered; https://apps.who.int/trialsearch/Trial2.aspx?TrialID=DRKS00018778.

**Supplementary Information:**

The online version contains supplementary material available at 10.1186/s13023-023-02739-w.

## Background

Children with medical complexity (CMC) have multiple chronic conditions and require long-term care accompanied by different health- and social care professionals [1]. The fragmentation of existing healthcare systems makes it difficult to obtain healthcare services for CMC, especially for children with rare diseases [2]. Spinal muscular atrophy (SMA) is a rare genetic, neuromuscular disease (incidence of 1:6.000 to 1:10.000 births/year) characterized by the degeneration of alpha motor neurons in the spinal cord due to loss or dysfunction of the SMN-1 gene 5q11-q13 [3]. The care of spinal muscular atrophy (SMA) involves several components. According to the “International Standards of Care for SMA”, in addition to drug therapy, supportive treatment measures are also part of the care of affected individuals [3, 4]. Despite significant improvements in pharmacological treatment SMA in recent years [5–10], it remains a complex and chronic condition for the majority of patients [11]. The present study focuses on patients with SMA I and SMA II, the most severe subtypes [3].

Depending on the chronic condition’s severity, caregivers spend a lot of time coordinating appointments, communicating between providers, clarifying social legal issues, and more [2]. Efficient care coordination is often listed as addressing this problem as well as ensuring the overall quality of care [1, 2, 12]. There are various definitions of care coordination [13, 14], with Walton et al. providing one that is tailored to rare diseases [15]. They describe care coordination as a collaboration of the care network (healthcare professionals, the patient and his/her family) within the care “to avoid duplication and achieve shared outcomes, throughout a person’s whole life, across all parts of the health and care system […]” (10, p. 8).

The path to optimal care coordination of rare diseases involves enabling factors and barriers that affect different areas. The first area covers the role distribution in the care network. For example, common care coordination models often propose a central coordinating point that bears the overall responsibility within care. However, it is unclear who exactly holds this position: healthcare professionals, patients or caregivers, administrative coordinators, etc. [15, 16]. The second area concerns relational factors within the care network, such as communication, information exchange, etc. [1, 15, 16]. This area includes, for example, the extent to which medical documents are shared within the care network, or the quality of relationships between care network members. Other peripheral aspects of care coordination relate to access to (specialized) care (e.g., availability of healthcare professionals with expertise in the specific condition) or existing characteristics of the family environment (e.g., family resources for disease or coordination management) [1, 15].

While previous studies have primarily examined care coordination in conjunction with frequent medical conditions, relatively few studies focus on rare conditions. Despite similar needs, coordination for rare diseases may involve additional components or components that differ in their delivery from care coordination in more widespread conditions [15]. For this reason, this study highlights caregiver’s experiences in the context of care coordination of children affected by SMA I or SMA II.

## Methods

### Study design and research aims

This study is part of the exploratory, prospective, controlled, two-armed SMA-C+ -study, developed as an IT-supported Case Management to improve the care of children with spinal muscular atrophy I and II [17]. The interviews conducted evaluated experiences with the children’s current care situation from the perspective of caregivers, as well as their tasks in care and care coordination. Results are reported using the consolidated criteria for reporting qualitative research (COREQ) checklist [18].

### Participants and recruiting

We interviewed N = 21 caregivers whose children have a genetically confirmed SMA type I or type II. Participation was independent of the length of the child's disease history, i.e. inclusion was possible immediately after diagnosis or at a later stage. Newborn screening for SMA was initiated in our recruitment district after recruitment was completed. We recruited caregivers if their children receive treatment in one of three neuromuscular centers in Germany participating in the study. Participants were selected using a maximum variation sampling approach (purposeful sampling) guided by contrasting characteristics, such as patient age, health condition/stage of illness, family situation, and geographic location. Potential participants were being continuously identified and recruited through personal contact with the treating neuropediatricians in the neuromuscular centers. Eligibility criteria were controlled during the recruitment process. All participating caregivers provided written informed consent to participate, including having their interview audiotaped and further processed. No participant withdrew his/her participation after having been invited.

### Interview guideline

The semi-structured interview guideline we followed was drafted by J.W. following Helfferich [19] and finalized after review by the whole team [see Additional file 1]. We relied on the “life-course theory” (LCT) as a theoretical framework in developing the interview guide [20]. The LCT is suitable because it focuses on predictable transitions in the lives of patients, the family context, and aspects of stress and resilience.

We divided the guideline into three obligatory main blocks on the topics (a) “General experiences in caring for a child diagnosed with SMA I or II”, (b) “Experiences in the care network”, (c) “Experiences with care coordination”. Each question block contained one to three open-ended obligatory core questions and several optional questions that subsumed the key aspects of each topic. Interviewees were allowed to freely report on their experiences; questions were only posed if participants did not propose relevant aspects of the core topics. Individual deviations from the wording and the order of the core questions were possible, and issues brought in by interviewees were followed up.

### Procedure and transcription

J.W., a psychologist with experience in counseling and conversation techniques, interviewed the participants individually between August 2019 and April 2020 using the developed guideline. The semi-structured interviews either took place at the SMA patient’s regular inpatient stay or were conducted via telephone. Apart from a short telephone contact to arrange the interview date, the interviewer did not know the interviewees beforehand. The interview was scheduled to last 60 min. All interviews were digitally audiotaped in full after receiving permission from the participants. The audio recordings were transcribed verbatim by an external transcription service provider, and personal data was pseudonymized. We did not return the transcripts to participants for comment or correction.

### Data analysis

We assessed the interviews’ transcripts via qualitative content analysis relying largely on the Kuckartz approach [21]. We took a deductive-inductive approach to create a code system. To ensure intersubjective comprehensibility, the coding frame and guideline were reviewed by I.B., T.L., and E.F. and adapted to their feedback. The multi-level procedure chosen for our study is outlined below: (a) We read intensively the transcribed text material in the process of pseudonymization and composed short case summaries. (b) We extracted codes inductively using the case summaries. In the next step, additional codes were derived deductively from key topics within the research question, the interview guideline and previous research on care coordination components [1, 15, 16, 22]. (c) We applied this initial coding frame to a quota sample consisting of 20% of the data material (N ≈ 5). During this process, the codes were refined several times through continuous reflection and classified into main and sub-codes. (d) We then started the first coding of the entire material along with the coding frame defined up until then. Codes were revised again if necessary, that is, they were summarized or differentiated into further sub-codes. (e) I.B. also intensively processed five randomly chosen interview transcripts based on the developed coding frame. Discrepancies in coding were discussed and the final set of 7 main codes and 12 sub-codes was defined by consensus (for an overview see Table 1). (f) This final coding system was applied to all transcripts by J.W. This process of (sub)code formation was iterative until an acceptable discriminatory power and depth of categories was achieved. (g) In the next step, we paraphrased all statements of a participant assigned to the same code. Overall findings were extracted from a code x participant-summary-matrix.

**Table 1:** Final coding system

Main codes	Sub-codes
Disease and coordination management of the caregivers	
General conditions of care	
Expertise and skills	Expertise and skills of HCP
	Expertise and skills of caregivers
Coordination structure	Existing coordination mechanisms
	Perceived coordination needs
Information exchange	Information exchange between caregivers and HCP
	Information exchange between caregivers
	(Perceived) information exchange between HCP
Role distribution in care coordination	Role of pediatrician
	Role of neuromuscular center
	Role of local care providers
	Role of SPC/early intervention center
	Role of caregivers
Quality of relationship	

Data were organized and analyzed using qualitative data analysis software MAXQDA Plus 2020 (version 20.0.3). Subsequent quantitative descriptive information was calculated with IBM SPSS Statistics (version 27). The entire patient data material to be analyzed took about 14 h and the single interviews lasted between 24 and 56 min. The interviews were conducted in German. We translated code descriptions and quotations taken from the interview into English.

## Results

### Sample

We interviewed 21 caregivers of children with SMA I and SMA II (characteristics detailed in Table 2).

**Table 2:** Caregiver and child characteristics (n = 21)

Variable	%
Respondent gender	
Female	71.4
Male	28.6
Respondent age	
< 30 years	9.5
30–40 years	57.2
41–50 years	33.3
Respondent education	
Primary school, secondary school and secondary modern	19.0
Completed training	23.8
University degree (Bachelor, Master, Doctorate)	28.6
Other	28.6
Respondent family status	
Single	4.8
Married	76.1
Living in a steady partnership	14.3
Divorced, separated	4.8
Respondent employment status	
Employee full-time	19.0
Employee part-time	38.1
Civil servant	4.8
Not gainfully employed or capable of gainful employment	33.3
Other	4.8
Child SMA diagnosis	
SMA I	62.0
SMA II	38.0
Child gender	
Female	47.6
Male	52.4
Child age at time of interview (years)	
2 years	14.3
3 years	19.0
4 years	33.3
5 years	4.8
7 years	9.5
11 years	14.3
15 years	4.8
Child age at time of diagnosis (months)	
1–4 months	23.8
5–12 months	47.6
13–18 months	23.8
> 18 months	4.8
Child age at drug therapy initiation	
< 4 months	9.5
5–11 months	28.6
1–3 years	33.3
4–10 years	23.8
> 11 years	4.8
Ventilatory support	47.6
Enteral nutrition	33.3
Cough assist	71.4
Best motor function achieved	
Rolling over without support	9.5
Crawling on hands and knees	28.6
Sits independently	28.6
Stands independently	9.5
None	23.8

### Disease and coordination management of the caregivers

This code describes the resources and barriers to coping with organizational or coordination-related disease demands and associated consequences. The caregivers interviewed reported four major topics in their disease and coordination management: (a) experiencing self-efficacy/confidence in coordinational/organizational tasks, (b) experiencing uncertainty/feelings of powerlessness in care coordination, (c) experiencing a sense of responsibility in care coordination, (d) dealing with the consequences of (perceived poor) care coordination. Depending on the topic, this code can benefit or detract from care coordination.

#### Experiencing self-efficacy/confidence in coordinational/organizational tasks

Only a few caregivers referred to this area. They reported the availability of a (social) network for support, e.g., parents, partners, or other affected persons. Similarly, they listed supportive sources of information, such as the Internet or meetings of patient advocacy organizations. They reported on their expertise growing through experience, giving them a sense of security concerning care coordination.

*The first one or two years were really up and down and nerve-wracking, but in the meantime we have everything well under control. We have our own nursing service. We have our therapists. Yes, we know very well what we ourselves have to do with infections, what we’ve got to inhale with, how we should react. We know when and how to give oxygen and which parameters on the pulse oximeter are good for him and which are bad. […] So yes, we already have a bit of medical expertise.*

*We were lucky that we are such an intact family that supports each other. We have a strong character and a solid education. We can assert ourselves well. But not everyone has such a background.*

#### Experiencing uncertainty/feelings of powerlessness in care coordination

Many more caregivers reported feelings of uncertainty and powerlessness concerning coordination of care. In this context, they mentioned the high level of information input, as well as a cognitive load due to a myriad of coordination tasks. This is associated with doubts about whether certain care options are the right ones or take place at the right time, as well as uncertainty about heterogeneous, symptomatic care in children with SMA.

*You sometimes feel very uncertain. It is quite good we have support groups between parents and doctors – but if opinions differ, then it is quite difficult to judge. Am I right? Or what should I do now?*

*What they’ve given me to read I can’t say anymore. That’s because there just too much input.*

#### Experiencing a sense of responsibility in care coordination

Closely related to the previous focus, nearly all caregivers reported a sense of (sole) responsibility within care coordination. The interviewees described the feeling of being permanently responsible for everything and feeling a great deal of pressure to act. Related to this, some of the caregivers interviewed reported that they felt they could not rely on healthcare professionals and therefore had to manage many issues themselves. They also described a high degree of motivation to inform themselves about SMA.

*We make sure that we also carry out therapy measures on him together with our nurses. Every day we do something, because we have noticed […] whatever you do, it’s not enough with SMA.*

*[…] we have become members of the German Society for Muscular Dystrophy. It is good to be able to exchange information. You simply see how other families manage it. That always gives you some incentive.*

*It's true, […] we have to invest a lot of time in […] passing on all the information and really taking care of every detail. Of course, it takes a lot of effort to think everything through. Sometimes you have a lot of respect for this responsibility… Sometimes it works out better when you face it, and sometimes it doesn’t.*

#### Dealing with the consequences of (perceived insufficient) care coordination

Furthermore, a few caregivers reported the consequences of (perceived insufficient) care coordination. Some treatments were initiated too late in the eyes of the caregivers. They described that it’s hard to take having to wait for them, and to accept that time and effort on their part won’t bring the desired progress. Some even described a changed personality because of intensive care coordination as well as insisting on delaying treatments (e.g., requesting aids).

*And it’s the same with this disease: The sooner you can start treatment, the sooner you’ll succeed. I would say that we already observed the first symptoms four to six weeks after birth. E. was five months old when we first got treatment. And that's very frustrating.*

*Well, I’ve also changed a lot as a result. I used to be very shy. I didn’t use to try to follow my own instinct or a feeling – I really just accepted what was being said. Nowadays I'm different.*

### General conditions of care

General care conditions include relatively permanent and persistent conditions with an impact on care coordination [23]. This mainly comprises organizational aspects guiding care provision from the care network and accompanying the family as they move through care [1]. Within this category, we have identified two different topics: (a) the availability and compatibility of (suitable) healthcare professionals, and (b), healthcare organization structures.

#### Availability and compatibility of (suitable) healthcare professionals

For patients with SMA, there are local care providers close to home (e.g., occupational therapy, physiotherapy, speech therapy, and home care service) who provide supportive therapy or nursing assistance. There are also specialized facilities (e.g., neuromuscular center, social pediatric center (SPC), etc.) that provide drug therapy along with other SMA-specific services, and are often located farther away.

A large proportion of caregivers interviewed reported a limited choice of (good) healthcare providers due to a sparse local care network. Most interviewees described the lack of local therapists and hospitals possessing sufficient expertise in this rare condition. Furthermore, there are few specialized neuromuscular centers nationwide. The caregivers reported long travel times and complicated coordination of their appointments. Many caregivers mentioned especially the long distance to the neuromuscular center, which makes it hard to manage SMA-specific problems close to home.

*Of course, it would be good if we were already connected to the SPC, because then you’re also in close proximity to everything. So [in the neuromuscular center] you’d get Spinraza treatment, but the SPC accompanies the whole development – that would be just ideal.*

*Exactly – we live in a rural area where the supply is generally worse. We keep trying to create a network somehow, but it is difficult. We've been at it for two years now.*

*The fact that the neuromuscular center is so far away from us makes things difficult – it’s 300 km. If something does not go well with S., we have no nearby neurologist we can call.*

*We live in a small town; there is no orthopedist with SMA patients in their file. One would tend to find something like that in a somewhat larger city, that is, I’ve got to travel a little further to get to those who often see an SMA patient in order to benefit from the knowledge that that therapist, that physician has.*

At the same time, some caregivers interviewed reported improved opportunities for coordination through relationships among healthcare professionals thanks to their local proximity.

*I should say that I come from V., where the doctors know each other quite well. They’ve already worked together since the diagnosis. That is perhaps an advantage of ours – that they know each other well and work together.*

#### Healthcare organization structures

Some interview participants criticized insufficient care coordination from specialists or local hospitals (e.g., long waiting times). This was particularly difficult in combination with strong pressure to act that caregivers felt regarding the coordination of their child’s care. Caregivers saw this as primarily due to limited time and human resources within the healthcare system.

*We waited six hours at our orthopedic appointment. We got very angry and asked them to ensure that that not happen again!*

*On the one hand, you go to see the specialist, but many other patients do that too. This makes things complicated: Two months waiting time for an appointment is quite normal.*

*But otherwise we do everything ourselves, because we know that staffing in the hospitals is simply too low. They can't put someone at M.'s bedside 24 h a day.*

A large proportion of respondents described intensive, repetitive contact with the health insurance company as a time-delaying component that can complicate care coordination further.

*The past two years were really stressful, especially because we had to convince our health insurance provider about what was right for our daughter.*

*There are the constant battles with the health insurance provider: they refuse to approve it. You have to do the paperwork again.*

*He had an SPIO body prescribed, which we rejected immediately. We filed an objection. And we haven't heard anything for a month […]. So I called the insurance again to ask what the situation is now. We’ve received no letter, nothing at all. And then you’re always asked to call back… and get contradiction after contradiction. And so far, everything we’ve asked to be approved has been initially rejected.*

### Expertise and skills

This code describes on the one hand the amount of the caregivers’ available expertise [1, 16]. On the other hand, caregivers’ statements regarding their skills (or shortage thereof) and the expertise (or lack thereof) of the healthcare professionals are summarized [1, 16]. Thereby, the term “skills” mainly refers to the medical field and their associated knowledge and not to meta-skills (e.g., good communication skills). Expertise, on the other hand, refers (beyond medical knowledge) to being informed about existing structures or existing rights/opportunities within the care of children with SMA. We divided this code into two sub-codes: (a) expertise and skills of HCP, (b) expertise and skills of caregivers.

#### Expertise and skills of HCP

A large proportion of interview participants described varying levels of general expertise about SMA. Due to the rarity of this disease, caregivers frequently reported limited knowledge on the part of healthcare professionals. In this context, caregivers also reported receiving varying levels of professional support, e.g., education after getting the diagnosis or when applying for aid, and information about new pharmacological treatments.

*I realize that some doctors know relatively little about this disease. We really noticed that last year when we saw the pediatrician and she said: “Oh, it’s a minor bronchitis; droplets are enough”. A few days later we were in the hospital with her intubated for the weekend.*

*Well, our pediatrician was more along the lines of: Now let everything sink in… but I couldn't sit idly by and do nothing. We became active ourselves because of that. Well, she does not have the […] knowledge about SMA, which is also because the disease is so rare. […] It's new territory for the doctors.*

*Why don't you find a good physical therapist for an SMA child? There is no such person! They just don’t exist. […] Specialized physiotherapists who know what they are doing: How far should I stretch this muscle? How far should I go? What about the muscle inflammation that occurs in SMA when you overstretch? That’s the kind of information that’s often lacking. The specialized knowledge is not there.*

In addition to disease-specific skills and knowledge, a small group of interview participants reported heterogeneous levels of knowledge about what additional services and opportunities exist in the care network (e.g., knowledge about the general existence of neuromuscular centers in Germany).

*What I missed in the hospital where we got the diagnosis is that we weren’t told that there are other hospitals with maybe more experience with SMA already, and that there are muscle centers in Germany. […] The doctor didn’t tell us he couldn’t help us, but perhaps the hospitals in Freiburg, Essen or Munich have more experience with this disease – and maybe it’s worth asking there. We found that out on our own.*

*For example, when doctors recommend an aid, they usually can't tell you where to obtain it.*

In addition, a few caregivers reported that healthcare professionals are also willing to communicate uncertainties about SMA and seek further education or information from specialized institutions or literature.

*Our pediatrician said right from the start that she had no experience, but that she’d be very happy to help us. She really gets information, and is in contact with another pediatrician who also has an SMA child. They exchange information. We have another hospital here in S that administers Spinraza, but they have no type I children. Our pediatrician is also in contact with that hospital and can get information there.*

*I'm thinking of our hospital here in our area, when they realize that they don’t know what to do anymore. Then they call the neuromuscular center because the (doctors there) know more about SMA who could help us. Yes, that’s what they communicate – that they don’t know what to do and will call the neuromuscular center.*

#### Expertise and skills of caregivers

This sub-code primarily describes the “growing into” an expert role reported by interview participants. A large proportion of caregivers described having to acquire knowledge about the disease and about care options. This expertise emerges primarily through experience and through networks such as the German Society for Muscular Dystrophy or a similar organization.

*But, as I said, hardly anyone knows anything about this. We’ve become SMA experts ourselves.*

*We now know that if a child has SMA and an infection, then he or she must in fact be ventilated. We did not know that at the beginning. It is all such a chain of events. Yes – now we’ve become smarter and know everything. But we didn’t know that at the time.*

*And above all, we parents managed to get organized in SMA Germany, in the German Society for Muscular Dystrophy. And we actually meet every year, also with the doctors. There we get information; you network very, very strongly with other parents and with the doctors who are there for the meeting. After that you’re on your own, and have to see that your child ultimately gets the right therapy.*

### Coordination structure

This code describes the importance of mechanisms explicitly designed to coordinate care for patients with SMA. We have divided it into (a) the description and evaluation of already existing coordination mechanisms as well as (b) the perceived need for new coordination mechanisms.

#### Existing coordination mechanisms

We used this sub-code for a caregiver-driven description and evaluation of the existing care coordination mechanisms of activities between the involved care network’s actors. These were both one-time and continuous coordination mechanisms. The largest component within this sub-code is the coordination of appointments. In this context, caregivers also reported on existing support in the coordination and organization of care (e.g. “aid consultation” at the SPC, assumption of tasks by the home care service). Some participants described the availability of an existing, ongoing point of contact (e.g., social services within the hospital) which coordinated appointments within the hospital.

*It’s an enormous relief for me that Mrs. W. organizes all the appointments. For example, if I say, next time I’d like to talk to the orthopedist; or three months before the appointment I say, we’re having stomach problems – then she organizes the appointments. I don’t have to call each doctor and ask for an appointment. If I need accommodation at the parents' residence at the neuromuscular center, she does that too. All we really need to do is say beforehand what the problems are, and she organizes everything for the next appointment.*

*Especially for aids – they have an employee who’s solely responsible for aids at the entire early intervention center. And she’s always there.*

The second major component within this sub-code is the description of structural links between healthcare professionals that enabled prompt coordination in the care network. In this context, caregivers reported on “connections” between healthcare professionals that facilitated, for example, enrollment in a hardship program or enabled home visits by a care provider. Only a few caregivers reported direct exchanges between care institutions.

*The neuropediatrician then confirmed it was SMA. And we told him that we already had an appointment in the neuromuscular center, so he then used his connections and had us register for this hardship program, because at that time the drug had not yet been officially approved.*

*I’d say that the only therapist who thinks and cooperates a bit more is the therapist at the kindergarten. For example, she provided us with contacts for therapists who come to our home.*

*The SPC made an appointment at the neuromuscular center for us. We came here after one month or even sooner for consultation. Then everything went really fast.*

*In some cases, this SPC already sends the prescriptions to the medical supply store. And the medical supply store then also submits them to the health insurance company.*

*L. needed a suction device when he was sick, and the pediatrician's office quickly clarified everything with the neuromuscular center. We got a prescription quickly, and the suction device arrived promptly.*

The last, less frequently mentioned component within the existing coordination mechanisms are linear, one-way information channels regarding SMA (e.g. a newsletter via e-mail). These serve to coordinate care primarily by bringing up previously unknown care options.

*And then there are various aid organizations and an SMA forum that regularly distribute news tickers by e-mail. And when you see how things look with the drug pipeline, the drug development phases, that’s quite helpful.*

#### Perceived coordination needs

This sub-code includes mechanisms perceived as necessary to coordinate care activities among the involved care network’s actors [1]. Many interviewees reported needing support or relief in (repeated) contact with individual care institutions or the health insurance company, e.g. to apply for aids, to establish a care network after diagnosis, or to coordinate appointments at the neuromuscular center. In this context, they mentioned the need for an “interface”/”bridge function” in the care network, as well as for care continuity [16, 24]. For better coordination, caregivers would like to have a designated contact person with medical expertise who supports them in medical discussions. Furthermore, they asked for professional exchange within the care network concerning their child’s care.

*Right after the diagnosis, when we were in F. for the first time, we knew very little about this disease. It might have been nice to simply provide information about this disease, what can happen, for example, that SMA children suffer more frequently from scoliosis, what the next steps are, how to proceed, what to do about certain things. For example, if the children are sick, that there’s something special to inhale. Just preventive things. We really missed getting such information. Instead, we got it all from our WhatsApp group.*

*If only there were someone I could always call and say: Mrs. or Mr. so-and-so, my child needs orthotics. That somebody will take care of that, that they’ll find the perfect company to take care of it – so that we don’t always have to wait 2–3 months until something gets done!*

*And that actually means two nights, two full days [inpatient stay at neuromuscular center]. What’s the other parent supposed to do? Where can you stay overnight? Where can you park? Are we entitled to any travel expenses? Being given a few tips would be great.*

*Maybe they could complement each other better – especially the occupational therapist who’s responsible for fine motor skills, and the physiotherapist makes sure that there are no contractures. If they exchanged more information, that would be even more effective.*

*It ‘s important to us that care distances be kept short. That I’m able ideally to combine appointments, where we can consult two or three doctors at the same time – that there’s close coordination, especially between orthopedics and neuropediatrics, as neuropediatricians are well informed about SMA, and orthopedists with the skeleton. We have not yet witnessed much cooperation between these two specialties. […] In orthopedics they always focus on orthopedic factors, and that’s it basically.*

*It would’ve been ideal had there been a single person who got to know L. from the beginning. That there’s one person with whom you exchange a lot and really trust. That there’s such a person who has an overview, knows what certain abilities L. has, how L. must be reclined.*

A few interviewees reported a need for more uniform standards of care. In this regard, caregivers would like to see coherent care recommendations from the healthcare professionals involved. One person mentioned the possibility of bundling information, e.g., a comprehensive reference book for SMA including socio-legal aspects.

*There is a lot of information you have to gather yourself. I’m involved with the patient advocacy organization and I don’t know of any document that contains everything you need to know. I mean, there are of course brochures, but there’s no checklist or guide in it: home healthcare check, degree of disability and so on. That would certainly be helpful at the beginning. Even if something like that exists, the question is whether it has been distributed or you still have to find the info yourself.*

*I have the impression, especially in the area of SMA, that there’s no guideline to follow somewhere, where one exchanges information from one orthopedist to another orthopedist. I think too much is done individually. I think that we should nevertheless more … not standardize, but we could learn more from each other.*

### Information exchange

This code describes the (non-)existing active, communicative and mutual exchange of information, ideas and opinions between the actors in the care network [1]. We distinguished three different sub-codes: (a) information exchange between caregivers and HCPs, (b) information exchange between caregivers, (c) (perceived) information exchange between HCPs.

#### Information exchange between caregivers and HCP

A large proportion of caregivers interviewed reported regular (or irregular) joint discussion of care options with healthcare professionals involved. These discussions included, on the one hand, care recommendations made by healthcare professionals. On the other hand, caregivers presented information to the healthcare professionals that they had obtained, for example, through previous exchanges with other affected persons on SMA-specific platforms.

*The exchange between the ward and intensive care unit worked well. In some cases there were round tables where we sat with I think five different senior physicians and therapists. Everyone was actually called in on a regular basis.*

*The wheelchair base is a standard frame and the seat shell on top is a special construction. The orthopedic technician got in touch with the wheelchair undercarriage builder and very often consulted with us and checked everything to make sure things were done properly.*

*We had situations where we had a hygiene problem […]. We didn’t want to leave the intensive care unit because things had happened in the normal ward that we did not feel were safe for A. The staff understood immediately, we met with the nursing management, with all kinds of people. We spoke openly about the incident so that everyone was on the same page.*

Many interviewees reported perceiving inconsistency or uncertainty in care recommendations from various healthcare professionals.

*There are differing opinions among different people. My child has problems with her hip. That means I have to sit down with four different doctors. One says this and the other says that. I don’t know myself what’s right or wrong.*

*We heard on the one hand the opinion that surgery was needed, whereas the other doctor said, as long as there’s no pain, we don’t need surgery. So we thought about it – how do we assess this situation now as parents? Which way do you go?*

*If you get the impression that a certain treatment did not go optimally or an aid doesn’t fit exactly, well, you’ll never get such a clear opinion from the experts: that’s bad and this is better. That’s more likely to come from other parents. But experts never confirm such information.*

*I took my child to the speech therapist and she gave me some tips because I had been so worried about my child’s eating, about swallowing. She said she thought we should thicken the drink. Then we had an appointment in the neuromuscular center with their speech therapist, and she said that it’s just the opposite, because the more you thicken it, the harder it is to swallow.*

In exchanging information, many caregivers emphasized the desire to be more involved in certain care processes (e.g., in debates about the approval of new drug therapy, production of individual aids).

*I think that you don’t get too little support, but too little info. Because of new medications, for example; that you don’t get enough information. That you also have to pursue it a bit […].*

*The interaction is not yet that clear to us. For example, with our daughter's corset: It was supposed to have a special corset shape. They said: “Your daughter won’t like it; she has to wear it 24 h a day.” Then we went to the orthopedic technician, and he said, “Not a corset like that after all, we’ll get another one, we’ll talk to orthopedics again.” Then suddenly something else came up, so it all passed us by without us ever getting the feeling we were involved or that we were being properly informed.*

*So I think it’s important that we as parents are talked to and that we stay in touch, because otherwise information gets stuck somewhere. As a parent I also want to stay in the conversation and be able to contribute to a better result.*

#### Information exchange between caregivers

This sub-code includes two major areas. First, the interviewees reported an information exchange regarding care which concerned care options, the quality of care institutions, secondary diseases, news in the field of SMA, etc. Furthermore, there was an exchange regarding daily life as well as the burdens associated with SMA. Many caregivers described mutual emotional support among affected families.

Channels of exchange included self-help groups or larger associations (e.g., conferences by the German Society for Muscular Dystrophy) and digital platforms (e.g., WhatsApp, Facebook) that ensure dynamic, two-way exchange among caregivers.

*I tried to talk to other parents to find out what they do with their kids, what everyday life is actually like.*

*We can learn more from each other and by that I mean it’s actually the parents among themselves: “Oh, I saw a great corset there!” “Oh, I went to rehab there and it was great for my kid!” The private, personal network – that’s actually how you get very far as well.*

*I’d become so desperate, and I turned to other mothers who, for example via Facebook, went public with their children, and they then helped me. Without such networks, we’d have gone under, because we’d never heard of a therapy chair or which aids are available at all.*

*And also the practical, everyday things – a lot comes from other families that you can’t get directly from the doctors or nurses because they’re not involved in your everyday life. That’s a very big part of the work – that you talk to other affected people and research everything yourself.*

*We’re always very happy when the information exchange takes place in M. This is done by the German Society for Muscular Dystrophy, and is sponsored by a large health insurance company in Germany. We soak up so much information in all the lectures over those two days. That’s actually the most important thing for us.*

#### (Perceived) information exchange between HCP

The interviewees reported a heterogeneous pattern regarding perceived information exchange among healthcare professionals. In some cases, caregivers reported a lack of collaboration among healthcare professionals from different disciplines involved in care. According to the caregivers interviewed, there should be recurring information exchanges and coordination on health status in terms of interdisciplinary care planning. Existing structural connections between the actors in the care network (including those in local proximity) would facilitate this exchange.

*That’s actually our major problem [the lack of communication between professionals]. Everyone does their own thing. We have no network. We pull strings everywhere, but somehow, nothing comes of it.*

*The communication between therapists and doctors and with us doesn’t really happen because here, we’ve only got locally our pediatrician, and she has no SMA expertise. […] If we’re worried about his physical condition, we contact the neuromuscular center beforehand and they examine him. But there’s is no exchange with our physiotherapist.*

*In general, we think a bit more exchange between therapists would be beneficial. Because if things come up linguistically in speech therapy, the other therapists can somehow take them into consideration. It would certainly be better if something like that were standard – that they somehow short-circuit each other every six months with the start of treatment and then perhaps once, twice a year […].*

*There are relatively short distances to get to physiotherapy and occupational therapy – they know each other. They’ve agreed that one will focus on fine motor skills, hands, and the upper body, while the other will concentrate more on sitting, walking, and working on the muscles in this area. They’re also coordinated, they also have a short commute, and they telephone. They also send brief reports to our family doctor so that he can issue a new prescription.*

Many respondents described the need for sharing caregivers' information with healthcare professionals within the care network. According to the participants interviewed, healthcare professionals should pass on information that falls outside the scope of their discipline so that caregivers need not repeat it during further consultations.

*The physiotherapist from the SPC said that she knows Mr. W. from the medical supply store, and she will call him and tell him about the shoes. That makes it easier, as the experts can talk to each other. But that’s an exception, I’d say.*

*I have to call the physiotherapist and ask if she’ll take care of it. Then I’ve got to call the health insurance company back. Then I’ve got to search for a company that will take care of it. I have to take everything into my own hands.*

*I told our pediatrician that I was worried he was gaining too much weight. Then he immediately contacted the neuromuscular center, and the nutritional counselor came, so the coordination and understanding have been great.*

*From what I’ve seen so far, there’s no direct communication between our local colleagues on site and those in the neuromuscular center. We bring the information from there and give it to our local doctors, or vice versa.*

A few respondents cited the digital SMA registry, including the SMArtCARE database [25], as a helpful element. These platforms collect recorded SMA-specific and make it accessible to healthcare professionals and those affected.

*In this portal, hospitals can access each other's therapy status. All the key data for Spinraza and therapies are stored there. And the hospitals can access it […] to see what the status quo of the children is.*

### Role distribution in care coordination

There are different roles reflecting varying coordinating responsibilities within an SMA patient’s care network. This code describes the caregivers' perception of these roles. This entails the attribution of responsibilities and tasks by caregivers to healthcare professionals, in the sense of “Who do I need for what?”; “Who is responsible for what and who should take over what?”. Respondents also described their own role in the care network. Overall, we divided the code into five sub-codes according to the most frequently mentioned actors within the care network: (a) role of the pediatrician, (b) the neuromuscular center’s role, (c) the role of local care providers, (d) role of SPC/early intervention center, and finally the (e) caregivers’ role.

#### Role of pediatrician

Since primary care is largely provided by pediatricians working in private practice, the pediatrician plays a key role. The pediatrician often serves as the first point of contact (especially at the time of diagnosis). However, the caregivers interviewed attributed various roles to their pediatricians: they can be divided into two components with potentially different effects on care coordination: First, approximately half of the caregivers interviewed described their pediatrician as a rather passive component in their child’s care. Because of their limited expertise, they would not rely on their pediatrician for SMA-specific questions, but would consult him/her mainly for acute infections (similar to healthy children). Some interview participants also described their pediatrician’s deliberate reticence on SMA-related issues, knowing that the neuromuscular center with more expertise was available. A few respondents reported their pediatrician as a sometimes delaying component in their care coordination.

*The pediatrician just signs the prescriptions I need. Apart from that, for example, the cardiologist called him to ask why he was sending S. for a cardiological check-up. He answered: “I don’t know”. I think to myself, "Didn't you even read your doctor's notes? My child had a hole in her heart when she was born. She had to be checked every six months because of her ventilation situation. So when it’s up to me to inform my pediatrician about what's going on… I can’t help but think – this can’t be true!*

*When the child is born and the first abnormalities are noticed in comparison to another child's development… That’s always played down, you’re portrayed a bit as a helicopter parent, but we should rely on the doctors and are told: “There’s nothing wrong.” Until you actually reach the point where things have become so drastic that not even the worst doctor in the village can ignore her symptoms.*

The second half of the interviewed participants perceived the pediatrician as a supportive component in care coordination. They described the pediatrician as a suitable contact thanks to being close by. A small proportion of caregivers reported that the pediatrician accommodated them, e.g., by issuing prescriptions, shortening waiting times, or making house calls.

*We have a good pediatrician who always helps me with everyday things. I think she’s the first point of contact.*

*Otherwise, the pediatrician is a great help to us because we call and say that we need a prescription for XY and he’ll say: “Come and pick it up tomorrow.” So that works very well, we're very satisfied.*

*I can talk openly with him about everything. Appointments are always made with less waiting time for D. and me. Sometimes I call back and ask how busy they are and he tells me: “Mrs. Z., you can come a little later.*

#### Role of neuromuscular center

Neuromuscular centers provide expert diagnosis and care for neuromuscular diseases, including SMA. They usually comprise neurologists or neuropediatricians who work in an interdisciplinary collaboration with cardiologists, pulmonologists, orthopedists, rheumatologists, physical therapists, and social counselors. Thanks to their high level of expertise in SMA, most caregivers interviewed described the neuromuscular center as their main point of contact within their child's care.

*When it comes to SMA, only the neuromuscular center is my contact.*

*When it comes to specific SMA information, the neuromuscular center is the place to go because they are simply the best informed. They know the disease and its course and the research.*

Because the neuromuscular center is usually a larger (university) hospital, the caregivers interviewed described associated advantages and disadvantages. Thanks to many highly specialized departments, SMA patients undergo comprehensive care in neuromuscular centers. At the same time, because of the sheer size of (university) hospitals, caregivers feared inadequate monitoring of their child's health status and care needs. A few caregivers reported inadequate information because of confusion about who is responsible for what. According to the participants interviewed, this had a negative impact on care coordination.

*That is what the doctors wanted, and it also suited us very well that you do not just give the medication, but also look at everything and have all the care in one hospital. Since then, things have been going quite well. We also do not see the need to change.*

*My nursing service was also upset and said: "That can't be real. We wrote down the points for them. We specifically told them to look for those. Why didn't they [the team of the neuromuscular center] do it?” Time is the problematic thing. They can't sort it out or coordinate it properly.*

#### Role of local care providers

This code summarizes the role played by local care providers. This includes all healthcare professionals involved in the supportive care of patients with SMA (e.g., physical therapy, occupational therapy, speech therapy, home care services, medical supply store/orthopedic technology, local hospitals). The keyword “local” refers to the proximity of the care providers to the homes of patients and their families. Because of local proximity and the resulting recurring (weekly) contact, a large proportion of caregivers interviewed described local care providers as “constant helpers” during the disease. The local care providers' continuously updated knowledge of the child's health status made them a beneficial component of care coordination. Many of the participants interviewed reported discussing appropriate therapies or aids together. A few interviewees reported feeling relieved by having to engage in fewer coordinational activities (e.g., phone calls to the health insurance company). Overall, according to most interviewees, local care providers filled the role of linking the neuromuscular center to the families. They conduct detailed reporting of inpatient stays and assist the caregivers in communicating with healthcare providers through their medical expertise.

*Our physiotherapist has been with us from the very beginning. She’s known us for three years now, so she’s known S. since she was six months old. I’d say she’s been with us through thick and thin.*

*The local therapists help us when we need aids. That’s very important.*

*Sometimes they [the nursing service] take things off my hands by simply making a quick phone call to the pediatrician if it’s about prescriptions or whatever.*

*We’ve got a very good medical supply store. We actually test everything in advance and if we get along with things well, I only have to get a prescription, submit it to them and they take care of everything else. They are also very competent.*

*The therapists who see her every week probably notice almost as much as I do. They’re more involved in everyday life than doctors or specialists.*

However, a few caregivers interviewed described the medical supply store in particular as a potential delaying factor within care coordination (e.g., because of manufacturing necessary aids inadequately or too late).

*Unfortunately, there are too few good medical supply stores – people who are really motivated and put their heart into helping the children. Sometimes you also need to use your imagination and a bit of fiddling around. You have to think a bit about the children and exchange ideas with caregivers.*

*We got a prescription for an adapted seat shell. We went to a medical supply store in our area and our daughter was measured. Then nothing happened for a month. I called them once: They still had nothing from the health insurance company. A few days later, the health insurance told me that they’d only gotten the request after I’d reminded the medical supply store. Suddenly the seat shell became a therapy chair. I called the medical supply store and they told me was the same thing. They then delivered a therapy chair without competent personnel who’d have been able to adjust it. Two weeks later the technician returned and found that the therapy chair was too big for our daughter.*

#### Role of SPC/early intervention center

Interdisciplinary social pediatric centers (SPC) provide care to children and adolescents with developmental disabilities and chronic conditions through diagnostic and/or therapeutic services. Early intervention centers are facilities providing education and/or medical-therapeutic assistance (usually including special therapies such as occupational therapy, physiotherapy, speech therapy, etc.) to children with disabilities during the first years of life. This code summarizes the role of both institutions since the caregivers interviewed associate the SPC and early intervention center with similar areas of responsibility in their children’s care context.

According to most of the caregivers we interviewed, both institutions served as contacts in establishing a local care network. They knew good local care providers near the caregivers' homes and could make recommendations regarding specialists for further treatment. Similar to local care providers, they relieved caregivers of coordinative tasks (e.g., taking over correspondence with the health insurance company; consultation about assisting devices). In particular, the SPC also provided a continuous point of contact for SMA-specific issues, as the neuromuscular center would often be harder to reach.

*This SPC sends some of the prescriptions to the medical supply store in advance.*

*For aids, we do everything through the early intervention center. They have tried different medical supply stores in the area and know which one is the best. They have an employee who only takes care of aids for the entire early intervention center. And she's always there.*

*We go to the SPC once a quarter for consultation on medical aids. They simply check: What is E’s current care level? For example, we got a corset a while ago. They check regularly: Does it still fit or need to be adjusted? Does a new one have to be prescribed? Of course, we can always start asking questions: Can we have care extended in general? For example, with a wheelchair or therapy chair for the home. About every six months, the SPC conducts a pediatric orthopedic consultation where someone from a hospital in Stuttgart comes and examines E’s bone structure and muscular development in general.*

Furthermore, a few respondents reported getting assistance from both institutions in terms of strengthening their disease management by, for example, assisting caregivers with therapy delivery at home or offering psychological support.

*The early intervention center is quite all right, the therapist was kind and showed me many things, how to deal with the D., how I can still do gymnastics with him at home, physio and stretching. He showed me everything.*

#### Role of caregivers

Caregivers play a significant role in the care coordination of their children with chronic conditions. All interview participants reported that they had an all-round perspective of their child's health. According to the caregivers we interviewed, this was associated with the coordination and organization of care that they sometimes managed exclusively on their own. By constantly monitoring their child’s health status, caregivers immediately notice changes. The interviewed participants made it clear to us that it was their responsibility to make the healthcare professionals aware of certain conditions. They reported acting as an information carrier, passing information between members of the care network.

*The flow of information has to take place somewhere and it always runs through us caregivers. When we’re in the hospital, we carry the information to the physios, to the medical supply store, to the nursing service. When A.'s disease state changed again or medications changed, I had to inform every single person.*

*I also have the feeling that as caregivers you have to see through everything and always know what to expect. The doctors also help, but even with ventilation control … I said, “I think the mask is already too small,” and then the doctors looked and found out: “Yes, unfortunately it is too small.” Had I not brought that up, it might not have been noticed so quickly. I believe that the caregivers see the children every day and notice everything much earlier than the experts, who only see the children on one day for an hour. The caregivers handle a lot on their own and observe themselves, and organize.*

*We often serve as the carrier of information, i.e. we carry the information from the physiotherapist to the orthopedist, from the orthopedist to the occupational therapist and to the SPC.*

*It all goes through us as a central intersection. That means my wife coordinates everything that comes into the house during the day. Everything that needs to be coordinated in the long term, I coordinate on the phone.*

*The caregivers are always asked how they see things, how they assess things; they’re already a bit of an expert.*

According to all respondents, a significant component of the caregivers' role was to autonomously research and organize care options. They did this primarily through the Internet, rehab- or SMA-specific events, and exchanges with other affected individuals. They reported evaluating care options they found, as well as seeking (second) opinions from healthcare professionals.

*I organize everything so that he has school support, coordinate appointments, send doctor's letters back or take to the examination that everything is available.*

*In the beginning we also had infections; with many hospital stays … We were unsure and did not have a cough assistant or a Pari Boy. These are all things we acquired ourselves. We sat down with other affected caregivers and they told us: “You need this and that.” There was nothing from the medical side. So we finally took care of things ourselves.*

*The doctor at the SPC knew what the disease was. What he did not know, because at that time it was still part of the hardship program, was what treatment options were available. We simply looked around on the Internet again: Who’s a specialist in this field, and what drug pipeline is there? That does lead to the goal, but it’s an insane search process, because you have to separate sense from nonsense very intensively.*

*We go to trade fairs ourselves. We go to the REHAB or the REHACARE. We go to professional lectures. We go to the SMA meeting once a year. We are affiliated with the German Society for Muscular Dystrophy. We are connected to the SMA initiative and to the children's hospice. We more or less have to provide the information. That rests on our shoulders. That's more or less our responsibility.*

In addition, a large proportion of caregivers described a generalized “keeping the ball rolling” to obtain certain care services (e.g., recurring phone calls to care institutions).

*We’re getting a new car seat. The approval for it came a long time ago, but no one informed me. Or like with our rehab buggy: please check whether we can get drum brakes for it, how much they cost, whether you should pay for them yourself or submit a request for them from the health insurance provider. […] I got no feedback on that either. You have to keep calling.*

*When the news came out that a drug was being delivered, we had to call the hospital four times to hear anything about it. It took four calls until the doctor finally called back.*

*That is a lot of phone calls. The diapers alone needed ten phone calls with the providers and health insurance until that went smoothly. It works relatively well when it’s rehearsed, but any new application is sheer horror. I spend about two hours a day on the phone alone for S.*

### Quality of relationship

Under this code, we subsume statements regarding the quality of the relationship between the healthcare professionals and the family as perceived by the caregivers [1]. It is the most represented code in terms of all transcripts considered. According to the interviewees, the focus is primarily on the available patient-centered (communication) skills of healthcare professionals. Caregivers indicated that their needs and feelings should be considered during interactions. A large proportion of participants interviewed emphasized a desire for continuity and coherence in the relationship. Providing “seamless” care would give them security in the context of care coordination. They demanded accessibility of health professionals; including regular contact and specialized knowledge, but also beyond (e.g., about the current developmental situation of the child).

*For five years we were always in the intensive care unit. For the past few months, we’ve been in a different ward. Which is OK, I feel more comfortable there. But the doctor who’s known us from the beginning, who’s also involved with the ventilation; he was on the new ward, but he no longer seemed interested in looking after my child. He’s the head physician. When I asked to see him, they’d say: “He has a lot to do. He’s got to do this and that.” But my child is just as important.*

*So his doctor, Dr. Y., is actually a specialist in this disease. And if he’s not there, I can’t ask anyone about anything, about the new medicine for example.*

*There won’t be any other hospital to consider for Spinraza. I know that Dr. X. has been injecting since last year in the children's hospital, but we don’t want to go there; and with H. it’s the same. In the neuromuscular center where we’ve been since the beginning, the doctors know him, he also knows the doctors and the nurses, and trusts them.*

*I know that Dr. Y. is always there and he always helps and if I have questions, I can call and always reach him. That gives me a sense of security – if something happens, I can also write him and ask something.*

*We’ve also had frequent contact with the head physician. He really listens to us, and updates us on the latest developments in medicine and new drugs. We always get good feedback, and the whole hospital and team we found very good. We feel we are in good hands.*

*They simply ascertained that my child now needed a PEG or ventilation. They failed to explain to us exactly why or how. I wish they were more sensitive to us caregivers and would explain exactly what the ventilation is for, and that it can help your child. Or that you can get support, maybe from a social service.*

The participants interviewed reported that they wanted their concerns to be taken seriously. In this case, the caregivers described a heterogeneous situation: In some healthcare-professional-family relationships, there would be a “trustful cooperation”. It means that the healthcare professionals rely on the caregivers' assessments. On the other hand, some interviewees reported relationships in which they had to defend their expert role regarding the health status of their child. They described the existence of “two parties” (families vs. healthcare professionals). The interview participants felt they had to stand up for their desired treatment methods or were confronted with decisions they had to accept.

*What’s still lacking is a bit more trust in us caregivers […]. A little more trust in us caregivers and perhaps also in our children. That would certainly help quite a bit.*

*That happens to us relatively often: Compared to the doctors and therapists, I spend much more time with my child. That means that I can actually judge his needs best, and I’m always grateful when the doctors accept suggestions from us. Unfortunately, in the initial phase, we actually experienced the opposite, as we weren’t taken completely seriously. In the meantime, we’ve found that most of them are aware that we’re very familiar with our child’s clinical condition.*

*And the doctor at that time didn’t want to prescribe ventilation for us, because he said: “If a child is ventilated at night, at some point he’ll have to be definitely ventilated 24 h.” Then it’s still up to caregivers how they want to handle it.*

*It is sometimes like that: You really have to fight for the life of your child. Because when they see that she has SMA, they’re already thinking: This child is going to die anyway. Why should we put ourselves out for that?*

*That depends on whether the doctor takes you seriously or not. Because some consider themselves to be “gods in white” – it doesn’t matter what you tell them; if they don’t know the answer, then you might as well be talking to a wall. On the other hand there are doctors who become alert and if they don’t know what to do, and they at least seek help.*

*It is always nice when the pediatricians rely a bit on us parents, which works for the most part with our pediatrician. That’s actually a certain kind of cooperation.*

*We went to the orthopedic consultation meeting in our SPC. There were two parties: my child and I, and opposite us, there were six or seven people from the SPC; the head physician, the orthopedist, some senior physician, a physiotherapist, someone from the local medical supply store, and one or two other people. I found this relationship strange, and we were confronted with certain information, it was like “take it or leave it!.*

## Discussion

This study examined the experiences of caregivers of children with SMA I or SMA II in the context of care coordination through a qualitative interview analysis. To our knowledge, this is the first study to explore the experience of care coordination in the context of SMA. Overall, we identified seven key areas that have an impact on care coordination: “Disease and coordination management of the caregivers”, “General conditions of care”, “Expertise and skills”, “Coordination structure”, “Information exchange”, “Role distribution”, and “Quality of the relationship between caregivers and HCP”. These categories emerged both based on previous studies of care coordination and inductively from statements made by interviewed participants. The categories chosen affect care coordination to varying degrees. While some categories seem directly related to care coordination, some seem to have a more peripheral impact. We intentionally did not split those areas into barriers and facilitating factors, as caregivers often reported a “mixed portrait”. As a result, each area can have a facilitating or a delaying effect on care coordination, depending on how it is framed.

According to the data, care coordination in SMA seems primarily the caregivers’ responsibility. Their “Disease and coordination management” can provide them with either security or uncertainty within their activities. Hence, this code provides a kind of basis on which caregivers can coordinate individual aspects in their child's care with varying degrees of efficiency. In general, participants interviewed reported feeling more confident in coordinating care as their experience increased regarding their child's medical condition and health status. Caregivers of older children, who have been diagnosed with SMA for a longer period, reported higher levels of “Expertise and skills” on average and gained routine in care coordination. They could better navigate the various care options and align individual care interventions, compared to the disease’s beginning. While caregivers with younger children reported higher turnover in the care network, those with older children described a more consolidated structure because they had “found the right people”. This led to a concretization of roles within the care network.

The composition of the child's care network varies depending on the families’ homes and the available expertise and skills of healthcare professionals. Healthcare professionals with a higher level of “Expertise and skills” regarding SMA can be a supportive factor within care coordination. They can guide families within care options and assist them in expanding the care network. In general, caregivers reported difficulty to find suitable healthcare professionals with sufficient expertise in SMA. In urban areas, there seems to be greater availability of specialists. According to the data, this enhanced care coordination because distances were shorter and caregivers could combine appointments. For patients with rare diseases, it can be additionally challenging to depend on a few specialized services that focus mainly on the biomedical aspects of research and treatment but have little information about their patients’ overall functioning and participation in different areas of life. Therefore, it is important that the healthcare provider’s expertise exceeds purely medical knowledge. Rather, it is about identifying the needs of affected families to improve health-related quality of life.

Caregivers also reported pre-existing coordination mechanisms of varying quality. These mechanisms were often embedded in organizational structures of care. According to our data, they, however, do not appear to be representative of care for children with SMA across the country but instead, vary from family to family depending on their care network.

An important aspect of care coordination is the collaboration among the care network’s members that is reflected especially in the last three codes. The theory of relational coordination, that supported the inductive-deductive code formation, is a framework for assessing teamwork that emphasizes communication and relationships in a team [1, 16, 22]. Communication and relationships are dependent, resulting in a mutually reinforcing process [26]. Accordingly, the concepts of the last three codes are interrelated concerning their impact on care coordination [1, 16]: The actors in the care network need timely, regular, and solution-oriented information exchange to perform their roles well. Sharing information is easier when there are solid relationships among care network members. Good quality relationships ensure that all actors are aware of their roles. The role of a healthcare professional seems to be more extensive, provided there is a good relationship with the family. There are also connections between the more framing codes (e.g., “Disease and coordination management of the caregivers”, “General conditions of care”, “Expertise and skills”) and the relational coordination codes. With a higher level of care network members’ “Expertise and skills”, there is also stronger role awareness. Information exchange is more successful if adequate (coordination) structures are in place. Adaptation of the care process is easier if the care network members collaborate and respect each other, thus increasing trust of the patient and his family (“Quality of the relationship between caregivers and HCP”).

Nevertheless, the relational coordination theory mainly focuses on collaboration between interprofessional healthcare professionals. A unique feature in the care of patients with SMA is the role of the caregivers described as an interface in the care network. Caregivers reported they often found themselves coordinating treatments and providing information to all care network members. When their child is in poorer health, they described a greater coordination effort, as more aids were needed or more healthcare services had to be accessed (= more local care providers in the care network). Individual caregivers reported more coordination work; in the case of two caregivers, they often split the coordination. According to our data, exchange between caregivers of different children seemed to be an important link between care networks. Particularly at the beginning of the disease, this exchange was considered helpful because caregivers with newly diagnosed children reported that they benefited from those with more experience.

It is mandatory that caregivers play an intensive role in care coordination, as they are experts on their own child’s disease course. This can apply to some but not all caregivers, particularly when conditions are complex [27, 28]. Consequently, all 21 caregivers interviewed indicated that they needed coordination support in at least some areas. All 21 caregivers reported that they would have liked more coordination support shortly after receiving the diagnosis.

A strength of this study is that we conducted in-depth interviews with caregivers nationwide to understand their care coordination activities in the context of their daily lives. We were thus able to query their individual coordination needs and incorporate them thematically, in a different way than might have been possible with a standardized quantitative instrument. Another strength is the layout of our interview guide that provided little structure so that interviewees could adequately reveal their own perspectives. Moreover, the combination of inductive and deductive categorization is an advantage. We ensured that we covered a large proportion of hindering and facilitating factors regarding care coordination.

The study also has some limitations. A clear theoretical framing of the extracted codes was complicated. Authors named components of care coordination differently although they may well have meant essentially the same thing. We have to assume that we cannot make a clear distinction between the codes because they intercorrelate. As SMA is such a rare condition, very few participants were eligible to participate in our study from the get-go. We were unable to include caregivers whose child had recently been diagnosed with SMA. Consequently, our sample of caregivers predominantly represents those experienced in caring for their child with SMA. Further, we needed to exclude caregivers with limited German proficiency, although they are more likely to experience even more challenges related to this research topic. We also included families with children being cared for in specialized academic neuromuscular centers. Although there are over 50 certified neuromuscular centers in Germany, they are not all specialized in the care of children and adolescents and not all are academic centers. Therefore, our sample may be biased. Furthermore, as part of our inclusion criteria, we interviewed only caregivers of children with SMA I and II. The severity of the disease is “positively associated with a higher duration of informal care” (31, p. 8). For this reason, it can be assumed that our sample’s care-coordination needs are higher than those of other SMA types. Nonetheless, the cutoffs between SMA types are fluid, and the need for care coordination does not exclusively depend on disease severity.

Since the organization of healthcare for children with special needs depends on country-specific factors, our results mainly apply to German patients and caregivers. Nonetheless, recent studies and systematic reviews have found that the burdens and informal care experienced by caregivers of children with SMA are similar across countries [29–31]. According to Brandt and colleagues, “parents spend much time and energy on closing information gaps and navigating through the healthcare and support system, trying to find suitable solutions for their child” in addition to the time-consuming daily care tasks (30, p. 19). Even though the agents in the care network may differ in other countries’ healthcare contexts, care-coordination models might be necessary across all contexts. We were able to discover starting points for coordination through our interviews, but we were unable to deduce which form of coordination is ideal. Coordination mechanisms already tested in the context of other chronic diseases (e.g., Case Management, Care Manager etc.) could also work for SMA.

Finally, we conducted our study prior to the authorization of disease-modifying therapy options for SMA. Brandt et al. emphasize that care network support becomes even more important under these circumstances [30]. Future research groups will need to examine the relevance of care coordination in light of novel drug therapies.

## Conclusion

**Fig. 1:**
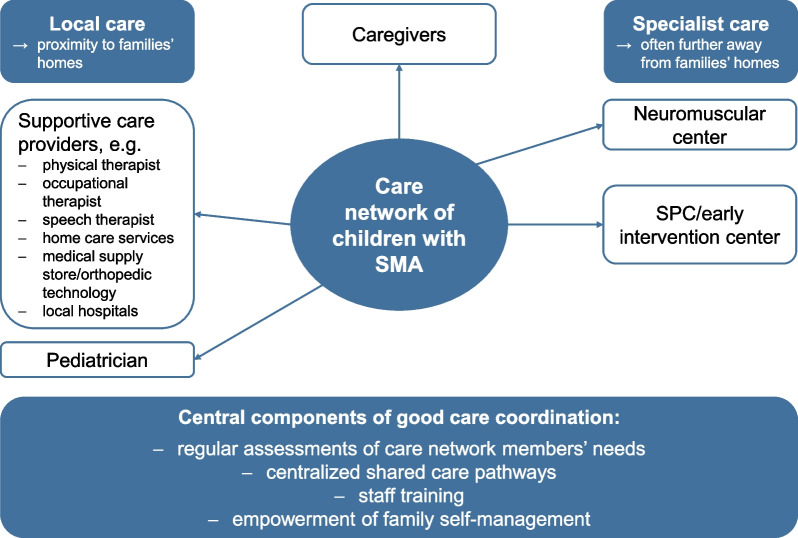
Template for a possible care network of children with SMA based on interviewees’ feedback

Overall, we conclude that it is probably impossible to make recommendations for care coordination that will accommodate the needs of all families with SMA children. Rather, coordination structures emerge depending on the family situation as well as the availability and constitution of the care network. Families often rely on coordinating care themselves, but their opportunities are limited. In general, despite the existing standard of care, the care of SMA patients is highly individualized and dynamic. There is evidence that we need an individualized approach to coordination for rare conditions based on differences in the extent to which families want to be involved [15]. However, our study has successfully documented good coordination within a care network of children with SMA that could function as a template to explain the potential care network to caregivers (Fig. 1).

## Supplementary Information

**Additional file 1.** Interview guide.pdf: Interview guide used in semi-structured interviews.

## Data Availability

Not applicable.

## Abbreviations

CMC: Children with medical complexity
HCP: Healthcare professionals
SMA: Spinal muscular atrophy
SPC: Social pediatric center
