# Supplementary material for: Barriers and facilitating factors of care coordination for children with spinal muscular atrophy type I and II from the caregivers' perspective: an interview study
Source: Orphanet J Rare Dis. 2023 Jun 2;18:136. doi: 10.1186/s13023-023-02739-w (PMC10239104; doi:10.1186/s13023-023-02739-w)
Supplement: Supplementary file 1 — Additional file 1. Interview guide.pdf: Interview guide used in semi-structured interviews. [file 13023_2023_2739_MOESM1_ESM.pdf]

Interview guide with caregivers

Introduction

- Hello Ms/Mr X, thank you for taking the time to do this interview with me today.
- My name is J.W., I am psychologist and work in the Section for Health Services Research and Rehabilitation Research, in short SEVERA, at the University Hospital Freiburg. We work closely with the Center for Pediatrics and Dr. Langer, with whom you had talked about the interview.
- Approximately 60 minutes are allotted for this interview.
- As you have already read in the information letter, this interview will be recorded by tape. If you mention names or other personal data, these will be deleted so that no one can draw any conclusions about you as a person. 36 months after the end of the project the complete tape recording will be deleted.

Today's interview is part of a research project that the Center for Pediatrics is conducting in cooperation with SEVERA.
The aim of this project is to improve the interaction between all people involved in the care of children with SMA I and II to increase the quality of care and relieve the burden on the affected families. To check whether the project leads to improvements, we first need an assessment of the current care situation as a basis for our planning. We rely on your experience so that we can depict this as accurately as possible.

General experiences in caring for a child diagnosed with SMA I or II

1. I would like to get a closer look into your everyday family life.
   Could you please tell me about a typical day of the week with your child, for example Monday? How would you describe it?

2. I am going to read you a passage now, and I would like you to give your opinion afterwards.
   "Children with SMA have several doctors and therapists who see and care for them regularly. However, care is not always as optimally coordinated as it should be. It is often the parents who ensure that information is passed between doctors and therapists - in many studies, parents of chronically ill children are also referred to as experts regarding their own child's health."
   What do you think?

Experiences in the care network

1. Could you please tell me all the healthcare professionals involved in your child's care?

2. What does it mean for you that different therapists and doctors care for your child?
   a. Optional: What do you think is going well? What do you think is not going well?

3. How would you describe the collaboration between the healthcare professionals and you?
    a. Optional: Do you feel that your concerns are being heard?
    b. Optional: Do you experience the various healthcare professionals involved as a coordinated "team"?

Experiences with the care coordination

1. What are your current responsibilities in caring for your child?
    a. Optional: Which tasks do you like to perform and which ones would you prefer someone else to do?

2. What would the perfect care for your child look like?
    a. Optional: Do you think there should be someone who keeps track of the "overall situation"? Which person could that be?

Final question and adoption

Is there anything left that you would like to mention?
Thank you very much for your participation and for talking so openly with me about everything, it is very valuable for us. All the best and a nice day to you.
